# Reconsidering the advisor–advisee relationship: Beyond the dichotomy between rigor and abuse in graduate education

**DOI:** 10.17179/excli2026-9428

**Published:** 2026-05-13

**Authors:** Jussara Secundo Dos Santos

**Affiliations:** 1 Graduate Program in Pharmaceutical Sciences, Federal University of Sergipe (UFS), Aracaju, Sergipe, Brazil

## ⁯

We read with interest the letter by Serafini and Quintans-Júnior (2026[5]) which reflects on the advisor-advisee relationship and raises the possibility that academic rigor may be misinterpreted as abuse. While this perspective contributes to an important discussion, a more nuanced interpretation appears warranted in light of the growing empirical literature on mental health among graduate students.

A consistent body of evidence indicates that postgraduate students, particularly doctoral researchers, experience elevated levels of psychological distress, including anxiety, depression, burnout, and suicidal ideation (Hazell et al., 2020[1]; Satinsky et al., 2021[4]). These outcomes cannot be fully accounted for by individual vulnerability or intrapsychic processes alone. Rather, they are closely associated with structural and relational dimensions of academic environments. In this context, the quality of supervision has repeatedly been identified as a central factor influencing student well-being (Hazell et al., 2020[1]; Mavrogalou-Foti et al., 2024[2]).

Supportive supervisory relationships, characterized by clarity, mutual respect, constructive feedback, and accessibility, are consistently associated with more favorable psychological outcomes. In contrast, environments marked by persistent ambiguity, insufficient support, or coercive dynamics have been linked to increased distress, emotional exhaustion, and adverse mental health outcomes (Satinsky et al., 2021[4]; Yao et al., 2023[6]). These findings suggest that supervision should be understood not only as a pedagogical process, but also as a key component of the psychosocial environment in which graduate training occurs.

It is important to acknowledge that graduate education is inherently demanding and involves exposure to critique, uncertainty, and revision. Such experiences are integral to academic development and should not be equated, in a simplistic manner, with harmful practices. However, the distinction between rigor and abuse is unlikely to be adequately captured by theoretical interpretations alone, nor by attributing students' responses primarily to individual psychological traits. Instead, it should be informed by empirical evidence, particularly with regard to supervisory behaviors, power asymmetries, and their measurable impact on mental health.

Recent findings further indicate that psychological distress in this population is reflected not only in self-reported symptoms, but also in treatment-related outcomes, including the use of psychotropic medications (Molina et al., 2025[3]). This observation reinforces the importance of examining how academic environments may contribute to, or help mitigate, such outcomes.

In this context, framing the debate as a tension between maintaining rigor and recognizing abuse may represent an overly simplified dichotomy. A more comprehensive perspective would acknowledge that academic rigor and psychologically sustainable environments are not mutually exclusive, but interdependent. Supervisors and institutions therefore play a central role not only in academic training, but also in fostering conditions that support student well-being.

Further empirical and interdisciplinary research is warranted to better understand these dynamics and to inform practices that promote both academic excellence and mental health in postgraduate education.

## Declaration

### Conflict of interest

The author declares no conflict of interest.

### Artificial Intelligence (AI) - assisted technology

AI-assisted tools were used exclusively for language refinement and structuring, without contribution to the scientific content.
